# Reproducibility Problems of Amyloid-β Self-Assembly and How to Deal With Them

**DOI:** 10.3389/fchem.2020.611227

**Published:** 2021-01-14

**Authors:** Peter Faller, Christelle Hureau

**Affiliations:** ^1^Institut de Chimie, UMR 7177, CNRS-Université de Strasbourg, Strasbourg, France; ^2^LCC-CNRS, Université de Toulouse, CNRS, Toulouse, France

**Keywords:** amyloid, peptide, reproducibility, self-assembly, aggregation

## Abstract

The self-assembly of peptides and proteins into amyloid fibrils and other aggregates are linked to several diseases. One of the most studied cases is the peptide amyloid-β (Aβ), found self-assembled in Alzheimer's disease patients' brains. In test tubes, assays with chemically synthesized or recombinant Aβ are widely investigated to understand the aggregation process and to find modulators, which could be of therapeutic interest. Experience over more than a decade in our laboratory through discussions with colleagues, expertly studying the literature, and as reviewers revealed to us the widely encountered difficulty to control the aggregation and obtain reproducible results in the test tube. However, this issue is scarcely reported and discussed in the publications, which we think hampers strongly the progress in this field and can deceive newcomers. Here, we describe the difficulty and potential reasons to obtain reproducible aggregation data and propose some guidelines for working with it.

## Introduction to Self-Assembly of Amyloidogenic Proteins

Amyloids are a central element in many diseases, including neurodegenerative ones. Peptides or proteins leading to amyloids include alpha-synuclein in Parkinson's disease and Amyloid-beta (Aβ) or tau in Alzheimer's disease (AD) (Chiti and Dobson, 2017; Törnquist et al., 2018). Amyloids with physiological roles are also known (Knowles et al., 2014).

Probably the most studied disease-related amyloidogenic peptide is Aβ due to its link with the highly prevalent AD. The Aβ peptide is the main constituent of the amyloid plaques, a hallmark of AD. In AD, Aβ and its variants are self-assembled under different aggregation mechanisms, including amorphous and fibrillar species. Similar high-molecular-weight aggregates can be obtained in the test tube by the self-assembly of monomeric Aβ. Many researchers have become interested in delineating the mechanism of the self-assembly process of Aβ into aggregates that mainly include amyloids (i.e., fibrillar) and amorphous species (Meisl et al., 2016).

Aβ is produced upon the enzymatic cleavage of the amyloid precursor protein. Several forms of Aβ can exist with different lengths and modifications (Kummer and Heneka, 2014). The two primary forms and most-studied ones are Aβ40 and Aβ42. Although Aβ40 is the most abundant, Aβ42 is more relevant to disease mechanisms as it is more prone to aggregate and accumulate in amyloid plaques (Hardy and Selkoe, 2002). Aβ40 and Aβ42 are present under healthy conditions at a low concentration in a monomeric state. Aggregates due to Aβ40 and Aβ42 self-assembly and fibril formation seem to be a pathological process linked to AD.

**Figure 1:**
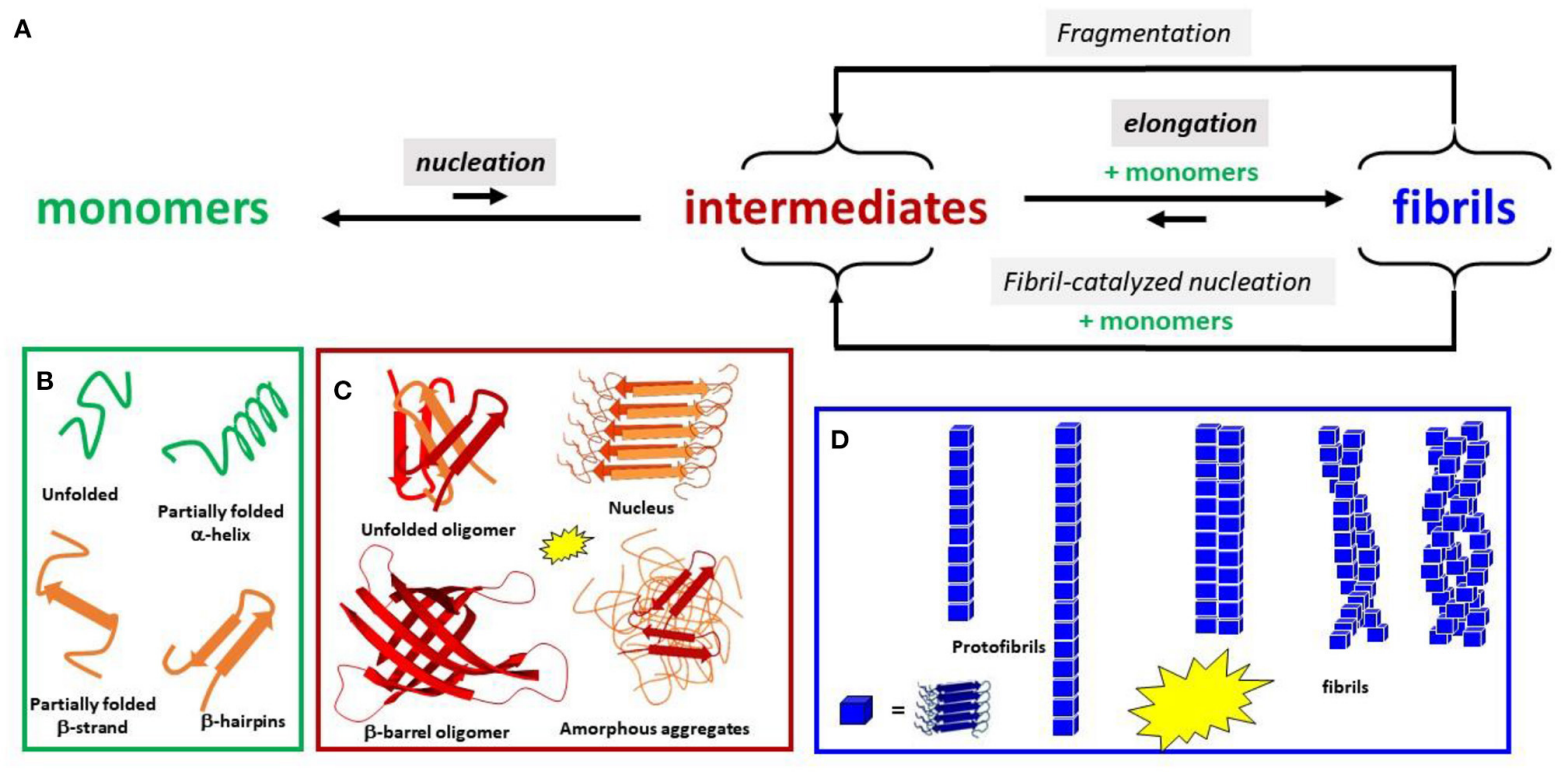
**(A)** Schematic view of the aggregation Aβ process. Only the main steps of the process are shown. Intensities of the fluorescence dye used as a probe of β-sheet content are shown by the yellow stars for the species formed. Several species are in equilibrium between each primary step: **(B)** monomeric conformers, **(C)** oligomers, and **(D)** fibrillary architectures.

In general, the self-assembly of peptides can lead to various aggregates that differ by several parameters (stability, size, global form, secondary structure elements, type and structure of interpeptidic interaction, etc. see Figure 1). The main purpose here is to illustrate and discuss the reproducibility of aggregation experiments in the test tube and propose ways to improve it. Thus, we briefly describe some key aspects of the mechanism of peptide aggregation (see below). If interested in aggregation mechanisms, the reader is invited to refer to recent and exhaustive reviews on that subject (see Kashchiev and Auer, 2010; Cerasoli et al., 2015; Nasica-Labouze et al., 2015; Chiti and Dobson, 2017; Stewart and Radford, 2017; Adamcik and Mezzenga, 2018; Srivastava et al., 2019).

In the present review, we use “aggregates” for any species made of more than one peptide regardless of its structure. It corresponds to mainly noncovalent polymers starting at dimers. Amyloids are fibrillar aggregates with a high content of cross-β sheet structure, organized in the forms of protofibrils and mature fibrils (made of several protofibrils) (Figure 1). The term “oligomers” is often ill-defined and is mainly used for “soluble” (i.e., not easy to sediment) aggregates with a limited size. Oligomers are now regarded as the most toxic species rather than fibrils (Hayden and Teplow, 2013; Lee et al., 2017; Rana and Sharma, 2019). Amorphous aggregates correspond to larger assemblies of mainly unstructured peptides.

Thermodynamically, amyloids are the most stable forms of any peptide/protein assembly; hence, they are more stable than monomers upon a critical concentration. The nucleus is, in contrast, the highest energy state and the least populated of all the forms during aggregation. It is the aggregate from which further association of monomers and small oligomers is faster than their dissociation. The size of the nucleus is not well defined with a lot of heterogeneity. Unlike the nuclei, oligomers are metastable species that can reach a certain level of population (Wetzel, 2006; Knowles et al., 2014).

The self-assembly process is macroscopically characterized by the lag phase, the growth phase, and the plateau, each corresponding to many elemental molecular events (Figure 1 and Supplementary Figure 1). Different aggregation states are simultaneously present, but their population evolves with time (Arosio et al., 2015; Meisl et al., 2016).

It is worth noting that, apart from primary nucleation (nucleus formation from monomers in solution), the secondary nucleation is an important event. Secondary nucleation includes (i) nucleation assisted by fibrils that serve as catalysts and (ii) fragmentation, by which several new nuclei are obtained from fibril splitting (see Figure 1). Secondary nucleation can be the main contribution to the growth phase (Cohen et al., 2013; Törnquist et al., 2018). Surface catalyzed nucleation is also important, where the surface could be biological membranes *in vivo* or the vessel and the air/water interface *in vitro*.

The kinetics of the fibrillation process are often reported by markers whose fluorescence turns on upon binding to β-sheet-rich assemblies. Thus, these markers are inappropriate to detect amorphous aggregates. The gold standard is the thioflavin-T (ThT) (Noël et al., 2013). Mathematically, the three aggregation phases are described by a sigmoidal curve (Equation 1), where *t*_1/2_ is the time required to reach half of the maximum fluorescence (*F*_max_) and *k* is the growth rate. The lag time *t*_*lag*_ is given by Equation (2). The various parameters depend on the rate of each individual aggregation step (Meisl et al., 2016).

(1) $$\begin{array}{l} F\left(t\right)=F_{0}+\;\frac{F_{\max}-F_{0}}{1+e^{-k\left(t-t_{1/2}\right)}} \end{array}$$

(2) $$\begin{array}{l} t_{lag}=\;t_{1/2}-\frac{2}{k} \end{array}$$

How the fibrillation curve depends on these parameters and other related equations are given in SI (Supplementary Figure 1). The *t*_1/2_ and *F*_max_ can be read on the curve, and the evaluation of *k* and *t*_*lag*_ requires simulation of the data. In addition to the kinetic parameters, the aggregation process can be defined by the morphology of the main final species as described by the average length, width, and distances between knots, if any, within the fibrils (Ke et al., 2017). External partners, including metal ions, lipids, carbohydrates, and proteins such as chaperons (etc.) that are often found colocalized with aggregated peptide deposits can significantly modify the peptide's aggregation process (Stewart and Radford, 2017; Ayala et al., 2019). Some external effectors, such as seeds (i.e., preformed fibrils), have also been used to increase the reproducibility (Giehm and Otzen, 2010). However, the reproducibility issue is then shifted to the production of the seed.

The aggregation process is challenging to study in the test tube. Below, we describe the current situation and propose some guidelines to improve it and overcome reproducibility issues that slow down amyloid-based research progress.

## Observations

### Introduction

For more than a decade, our groups have experienced difficulty getting reproducible results for the aggregation of the Aβ peptides that are probably one of the most studied peptides in life science. In the literature, when the same conditions are used (including in the same group), reproducibility is rarely reached. It even happens quite often that the opposite results are observed. For example, the addition of Cu(II) accelerated or slowed down fibrillation and increased or decreased the number of amyloids formed (reviewed in Viles, 2012; Faller et al., 2013).

One key reason is linked to the secondary nucleation process, which makes fibril formation an auto-catalytic process susceptible to external stimuli (Viles, 2012; Ke et al., 2017; Adamcik and Mezzenga, 2018). Also, the aggregation is a heterogeneous process with different possible paths and structures present simultaneously (Arosio et al., 2015). Thus, even unnoticeably different conditions can induce large changes in kinetics, contents, and morphologies of the formed aggregates (Hellstrand et al., 2010).

The most problematic part is that this issue is not documented well enough in the available literature. Reproducibility problems have been documented several times in the field of biomedical science (Prinz et al., 2011). However, this is rare for physicochemical research such as the self-assembly of peptides in the test tube herein discussed (Bergman and Danheiser, 2016).

### The Major Problem: The “Perfect” Monomerization of the Starting Peptide Sample

The sample preparation is crucial. The best way to maximize the reproducibility is to start with a 100% monomeric peptide because the presence of only one aggregate, regardless of its type, can have a drastic impact. Because an ideal sample of 100% monomeric peptide would reach a fast equilibrium with aggregates under conditions in which aggregation occurs, monomerization and storage of monomers have to be performed under nonaggregating conditions. Such conditions include at high pH, in an organic solvent, or in the presence of chaotropic agents. The aggregation event can further be triggered by changing to different conditions (pH and solvent). As a general feature, the number of preaggregated species depends on the peptide batch (hence, dependent on the synthesis, purification, and lyophilization processes). Being able to perfectly monomerize the peptide should eliminate the so-called “batch dependence.” This has been rarely achieved although several procedures (high pH, organic solvent, and chaotropic agents) toward this goal are found in the literature (Jan et al., 2010; Stine et al., 2011). Anyway, it seems that neither these treatments nor combinations of them solve the problem completely although it helps. A necessary further step is isolating the monomers under nonaggregative conditions (e.g., high pH), best performed by size-exclusion chromatography (SEC).

It is also possible to use a nucleation procedure, meaning that a preformed aggregate is used to promote the aggregation by adding a nucleus to template aggregation. However, this would only lead to reproducible results when the preformed aggregate solution is always the same, i.e., the same amount of each type of aggregation state is present. Although this is principally possible, it is complicated to prove due to the dynamic nature of aggregates and equilibrium between different aggregation states.

### Batch Dependence

The batch dependence was apparent to us by talking to other groups and by acting as peer-reviewers. For instance, some batches are toxic against cells, and some others are not. Similarly, although some batches form faster aggregates with Cu(II) or are inhibited by a “drug,” some other batches may not behave identically. The main discrepancy observed between batches is the time required to aggregate (in the same conditions). To overcome this issue, research groups may be tempted to choose the batch with the wanted property, a pragmatic but questionable approach.

### Illustration of Reproducibility Issues With In-house Aβ40 Aggregation Data

**Figure 2:**
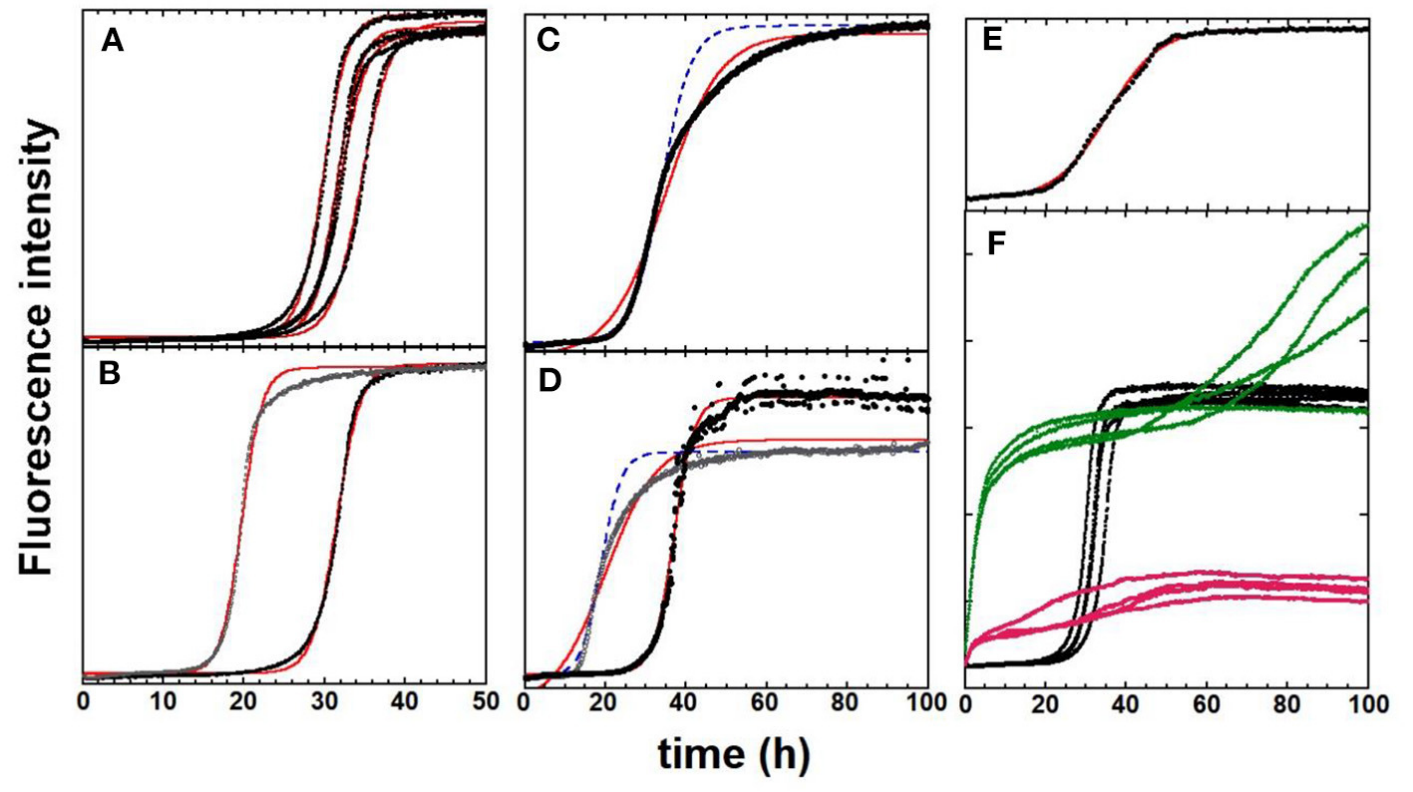
ThT fluorescence experiments to monitor the kinetics of Aβ40 aggregation in black [fits according to Equation (1) are in red. In some cases, curves obtained with manual adjustment of parameters are shown in dashed blue lines]. **(A)** The “ideal” case with four replicates in parallel from the same batch 1. **(B)** Impact of size-exclusion-chromatography fractioning. The two representative traces come from two fractions from the central part of the chromatography peak of batch 1. **(A,C,E)** Impact of peptide batch. Identical experimental conditions were used for three different batches. The s-shape curve is sigmoidal (**A**, batch 1), asymmetric (**C**, batch 2), and doubly sigmoidal (**E**, batch 3). The fits and manual reproduction of the experimental curves are not satisfactory for batches 2 and 3. **(D)** Impact of identical experimental protocol repetition with the same batch: Batch 4, but performed on different days (black vs. gray dots). **(F)** Impact of the addition of metal ions. Apo-Aβ40 (batch 1, black dots) with one mol equivalence of Zn(II) added (green) and with one mol equivalence of Cu(II) added (pink). The fluorescence values of all curves are directly comparable to each other. General conditions: Aβ40 at 20 μM in 50 mM Hepes buffer, pH 7.4. [ThT] = 10 μM. Except for **(A,F)**, in which one representative of each replicate is shown while all the replicates are given in SI with parameters deduced from the fitted curves. Data are in-house data unpublished except for **(A,F)**, from Conte-Daban et al. (2018).

The classical reproducibility issues encountered with Aβ aggregation experiments are illustrated in Figure 2 and are sequentially detailed below. Note that all the data shown here were obtained with an Aβ40 after a combination of high pH, chaotropic, and SEC monomerization treatments. Parameters to assess the reproducibility of the aggregation kinetics are (i) the ThT fluorescence value at the plateau (*F*_max_), (ii) the time needed to reach half of the *F*_max_ value (*t*_1/2_), and (iii) the shape of the sigmoid (steepness defined by *k*) (see Equation 1).

#### Reproducibility Between Replicates

Aggregation experiments are generally performed in multiplate fluorimeters, meaning that several replicates can be recorded simultaneously. The plate approach also allows for the simultaneous screening of several environmental conditions to question the aggregation process or test potential modulators of aggregation. Figure 2A shows a quadruplicate of Aβ40 aggregation curves, which are close to the ideal case. Indeed, all curves show the same trend (similar *F*_max_ and *k* values and a weak dispersion of the *t*_1/2_ values). However, it happens (fairly often) that, even though all data were collected precisely in the same manner, some curves are very different among the *n* replicates, leading to outliers (Supplementary Figure 2 for an illustration). Hence, when possible, the number of replicates should be at least three.

#### Impact of Monomerization

As previously stated, optimal monomerization is a crucial step for the study of Aβ self-assembly. In Figure 2B (and Supplementary Figure 3), the aggregation curves from two adjacent SEC fractions of the main chromatographic purification illustrate this difficulty. The two representative curves differ by two parameters. First, their *t*_1/2_, which is shorter for the first eluting SEC fraction in line with the possible higher contamination with small oligomers, and second, their shape. Although the curve obtained with the second elution fraction was almost perfectly fitted using Equation (1), this was less the case for the first eluting fraction (see Supplementary Figure 3).

#### Reproducibility Between Batches

Reproducibility between batches is the most complicated issue to overcome. This issue is illustrated in Figures 2A,C,E, in which the curves of the three batches acquired from the same company (ordered together and studied in the same experiment) are shown. The representative curves of the three batches (1, 2, and 3) are shown to illustrate the different possible shapes of aggregation curves. The aggregation curve of batch 1 (Figure 2A) is almost a perfect sigmoid, and those of batches 2 and 3 exhibit asymmetric (batch 2, Figure 2C) and doubly sigmoidal shapes (batch 3, Figure 2E), respectively (see Supplementary Figures 4, 5 for the fitting of data of batch 3). An asymmetric aggregation curve (batch 2, Figure 2B) mirrors a time-dependent growth rate *k*. Then, other fitting equations might be used (Shoffner and Schnell, 2016).

#### Reproducibility Between Identical Experiments

The issue of reproducibility is paramount, especially when encountered in identical experiments in which the same batch, experimental conditions, equipment, and investigator still leads to disparity in the measured kinetics. To illustrate that point, we show in Figure 2D, two experiments performed with the identical experimental setup and the same peptide batch but performed on different days. Differences are observed between the shape and *t*_1/2_ with the faster aggregation being described by an asymmetric s-shape curve. Such a situation is, however, one of the worst we have experienced. It is worth noting that, for asymmetric curves, such as those of Figures 2C,D, manual adjustment of the kinetic parameters in Equation (1) to reproduce the kinetic curve can be an alternative (dashed blue lines) because it is possible to model the first half of the curve, which gives access to the *t*_*lag*_, a key parameter.

#### Impact of External Modulator

Apart from these intrinsic reproducibility issues, adding external modulators can either improve or weaken the reproducibility. In Figure 2F, the effect of the equimolar ratio of copper or zinc ions is shown. Both metal ions do change the kinetics of the aggregation leading to a two-step process, in which the first step is swift, leading to a significant (zinc) or moderate (copper) enhancement of the ThT fluorescence and a second quasi-sigmoidal process, which can be reproduced using Equation (1) (see example in Supplementary Figure 6). Compared to the parent aggregation curve of apo-peptide from batch 1 (Figure 2A), the replicate is less similar in the presence of metal ions.

#### Other Sources of Irreproducibility

There are other possible sources of variation between results. Different investigators can cause issues as the handling of samples differs from one person to another even with stringent protocols. Other sources of irreproducibility include metallic contamination (mainly from buffer), different consumables (low-binding material should be preferred), and different spectrometers in link with the stirring protocol.

#### Conclusion

Thus, we conclude that there are two inherently essential questions linked with the reproducibility of amyloid aggregation in test tubes. First, when is it reasonable to consider that the effect of an aggregation modulator is significant? Second, how do we compare the aggregation process of different variants (e.g., mutants) of an amyloidogenic peptide? The latter question is mainly linked to the batch-dependence issue. We have strived to answer these questions in the discussion herein.

## Proposed Guidelines

Below we make some suggestions on how to address the reproducibility issue for experimentalists, supervisors, and peer-reviewers.

### For Researchers

#### Several Batches, Experiments, Replicates, and Investigators

We recommend using more than one peptide batch for the experiments (if possible from different sources and companies) and also conducting more than one experimental approach. A high number of replicates is also required to identify outliers clearly (Hellstrand et al., 2010). If the results obtained are statistically significant and batch- and experiment-independent, one can consider them as robust. These rules apply for comparison between different sequences or variants and the study of modulators of aggregation. It is also useful in a group that different persons repeat experiments. Even better would be that experimental results are confirmed by two different persons in separate groups, but this is often difficult.

#### Sequence and Purity of the Aβ

The purity and sequence of the Aβ have to be carefully checked. Purchased Aβ purity is based on HPLC and low-resolution mass spectrometry. Hence, anything not absorbing at 220 nm is not detected. This is also true for inversion of amino acid residues in the peptide sequence, for racemization of amino acid residues, and error in amino acid residues with the replacement of the desired amino acids by another one of close molecular weight (such as Asp to Asn, Glu to Gln, free C-terminus to amidated one and vice versa). Access to NMR or high-resolution MS with tandem MS/MS can help confirm that the ordered sequence was delivered.

#### Monomerization Procedure

It is essential to follow a detailed and rigorous procedure, including pre-monomerization and isolation of the fractions containing monomers using SEC. Treatments to monomerize pre-aggregates include the use of solvents (such as hexafluoroisopropanol), changes in pH far away from the pI (for Aβ at high pH), and chaotropic agents (Barrow et al., 1992; Fezoui et al., 2000; Hellstrand et al., 2010; Jan et al., 2010).

#### Other Handling Factors

Several cautions do help to enhance reproducibility. They include no useless handling that could introduce contaminants; storage of the peptide solutions at low temperature; and the use of low-binding tips, vials, and plates (Hellstrand et al., 2010).

#### Experimental Conditions

For some amyloidogenic peptides, triggering aggregation by an effector might help (Giehm and Otzen, 2010). Performing concentration-dependent experiments that allow the determination of the kinetics of elemental events can also secure the peptide's aggregation behavior. Indeed the *t*_1/2_ is expected to decrease while *F*_max_ and *k* are expected to increase as a function of the peptide concentration. Thus, experiments at several concentrations help to increase the robustness of the results (Meisl et al., 2016).

It is also worth being sure that the ThT concentration is not limiting as a deficiency in ThT modifies the shape of the aggregation curve, and a plateau is reached before the aggregation is finished because all ThT has been consumed.

#### Reports on Results

The central point is that, even if the aggregation experiments are not reproducible, it is important to report all the results obtained. Otherwise, if a preselection of batches has been made, the selection criteria must be reported. In line with the previous point, the pre-reproducibility concept is of utmost importance (Stark, 2018). Hence, it is worth documenting with as many details as possible all the pretreatments done before the experiments (amount, volumes, times, how the concentration of Aβ was determined) and the conditions of the aggregation experiment itself. The analysis of the curves must be well documented: mainly if and why some curves have been ruled out.

### For Referees

- They should check that the authors report on the points above, in particular, the use of several batches and detailed documentation.
- They should also be indulgent in knowing that variations between identical experiments and batches occur. Sometimes robust qualitative results are already promising results and certainly better than statistically significant results based on a nondocumented selection of batches.

## Data Availability Statement

The original contributions presented in the study are included in the article/Supplementary Materials, further inquiries can be directed to the corresponding author/s.

## Author Contributions

Both authors contributed to the writing of this perspective.

## Conflict of Interest

The authors declare that the research was conducted in the absence of any commercial or financial relationships that could be construed as a potential conflict of interest.
