# Supplementary material for: Reproducibility Problems of Amyloid-β Self-Assembly and How to Deal With Them
Source: Front Chem. 2021 Jan 14;8:611227. doi: 10.3389/fchem.2020.611227 (PMC7841044; doi:10.3389/fchem.2020.611227)
Supplement: Supplementary file 1 [file Table_1.DOCX]

Supplementary Material

# Supplementary Figures

# A.-sigmoid curve


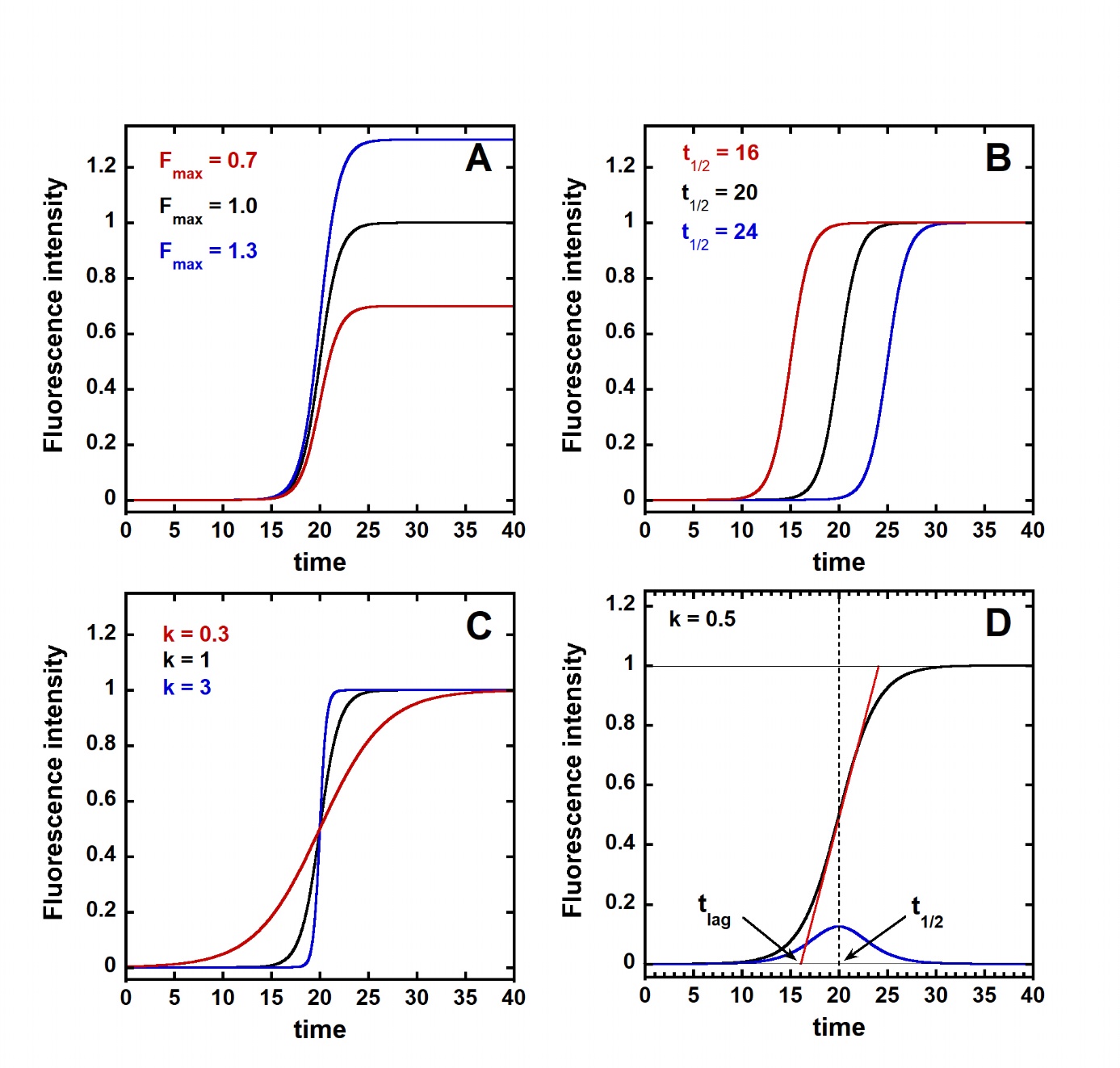


**Supplementary Figure S1.** Panels A to C: Illustration of the importance of various parameters in the sigmoid curve. Unless specified the parameters are: ΔF = 1 (F_0_ = 0, F_max_ = 1), k = 1, and t_1/2_ = 20. Panel D: A theoretical sigmoid curve (black line) and its derivative (blue line). The graphical measurement of t_1/2_ and t_lag_ are also shown, where t_1/2_ is the time required to reach half of the maximum fluorescnce (here 0.5) and t_lag_ corresponds to the intersection of the y = 0 line with the tangent of the sigmoid curve at t = t_1/2_.

Useful equations related to the sigmoid curve given by

- $F\left( t \right)=F_{0}+ \frac{\Delta F}{\left( 1+e^{-k\left( t-t_{1/2} \right)} \right)}$, with $\Delta F= F_{max}-F_{O}$ **(eqs 1.1)**

- $t_{lag}= t_{1/2}-\frac{2}{k}$, **(eq. 1.2)**

where $t_{lag}$ corresponds to the intersection of the y = 0 line with the tangent of the sigmoid curve at t =$t_{1/2}$.

- $F^{'}\left( t_{\frac{1}{2}} \right)= F_{max}^{'}= \frac{{kF}_{max}}{4}$ **(eq. 1.3)**

- $l= \frac{ln\left( 3-2\sqrt{2} \right)}{k}$, where $l$ is the half-width at maximum height of $F^{'}\left( t \right)$. **(eq. 1.4)**


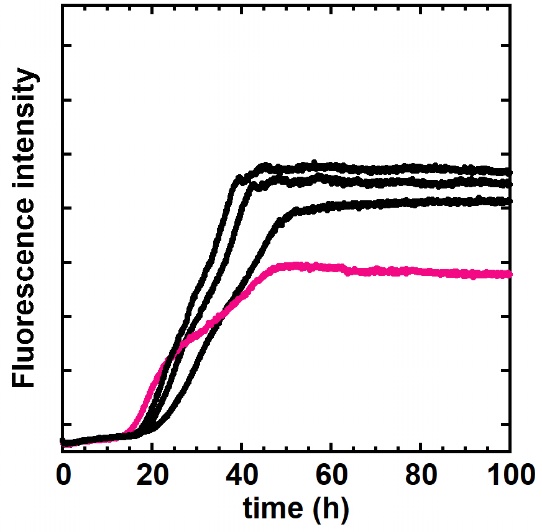


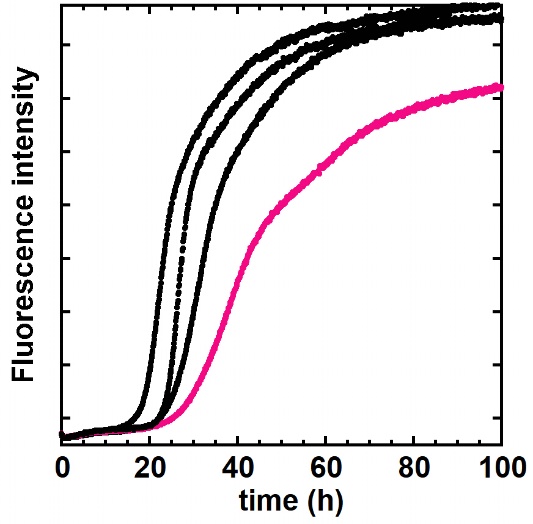


**Supplementary Figure S2.** All replicates corresponding to data shown in Figure 1, panels C and E. The “outliers” data are shown in pink.


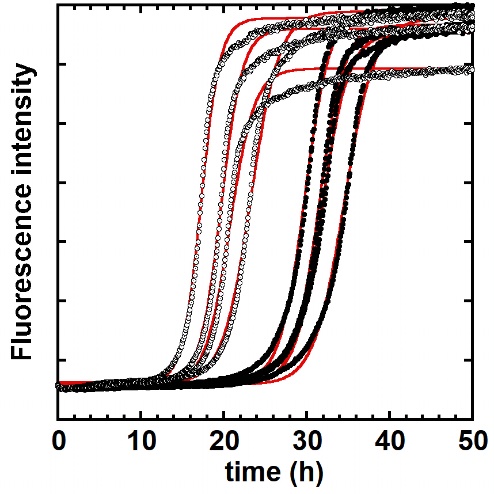


**Supplementary Figure S3.** All replicates corresponding to data shown in Figure 2, panels A and B, and corresponding fits using equation **1.1** leading to the following parameters given:

(second eluting fraction) $k=$ *0.62 ± 0.06 ;* $t_{\frac{1}{2}}$ *= 31.9 ± 2.0 ;* $F_{0}$*= 300 ± 8,* $F_{max}$ *= 3360 ± 85 ; GoF =0.9996 and* (first eluting fraction)$k=$ *0.73 ± 0.13 ;* $t_{1/2}$ *= 20.3 ± 2.5 ;* $F_{0}$*= 295 ± 9,* $F_{max}$ *= 3270 ± 215 ; GoF =0.9986.*

**B. Double sigmoidal curves.**

There is a last classical case where the ThT fluorescence curve are the superimposition of two s-shape curve (Panels E and F).

In that case, the fluorescence can be modelled according to:

$F\left( t \right)=F_{0}+ \frac{{\Delta F}^{1}}{\left( 1+e^{-k^{1}\left( t-t_{1/2}^{1} \right)} \right)}+ \frac{{\Delta F}^{2}}{\left( 1+e^{-k^{2}\left( t-t_{1/2}^{2} \right)} \right)}$ **(eq. 3.1)**


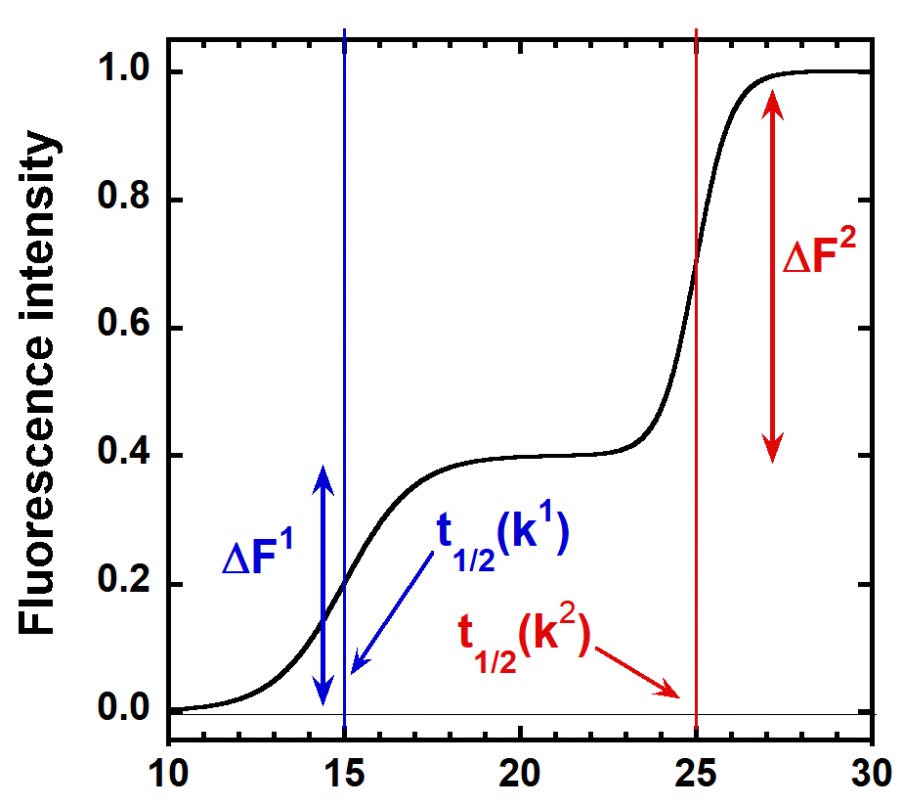


**Supplementary Figure S4:** Illustration of the importance of various parameters in the double sigmoid shape, with parameters defined in equation (3.1).


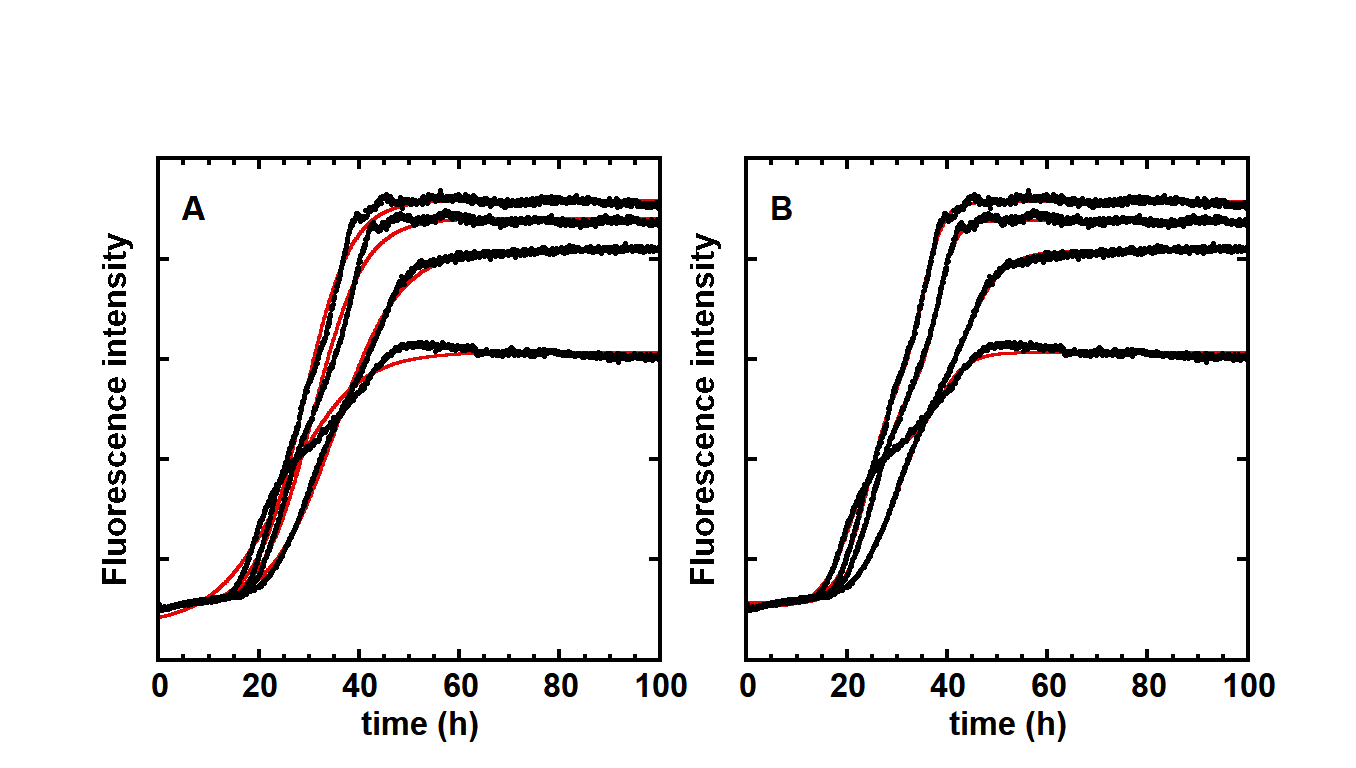


**Supplementary Figure S5.** All replicates corresponding to data shown in Figure 1, panel E and corresponding fits using equation 1.1 (left) leading to *(*$k=$ 0.18 ± 0.03 *;* $t_{1/2}$ = 29.9 ± 4.2 *;* $F_{0}$*=* 236 ± 39*,* $F_{max}$ *=* 2021 ± 338 *;* GoF =0.9981 ± 0.0016) or equation 3.1 (right)leading to *(*$k^{1}=$ 0.317 ± 0.04 *;* $t_{1/2}^{1}$ = 24.8 ± 3.7 *;* $F_{0}^{1}$*=* 276 ± 8*,* $F_{max}^{1}$ *=* 1298 ± 171 *;* $k^{2}=$ 0.417 ± 0.13 *;* $t_{1/2}^{2}$ = 38.8 ± 3.5 *;* $F_{0}^{2}$*=* 1086 ± 166*,* $F_{max}^{2}$ *=* 1801 ± 212; GoF =0.99962 ± 0.00039*.*).

*
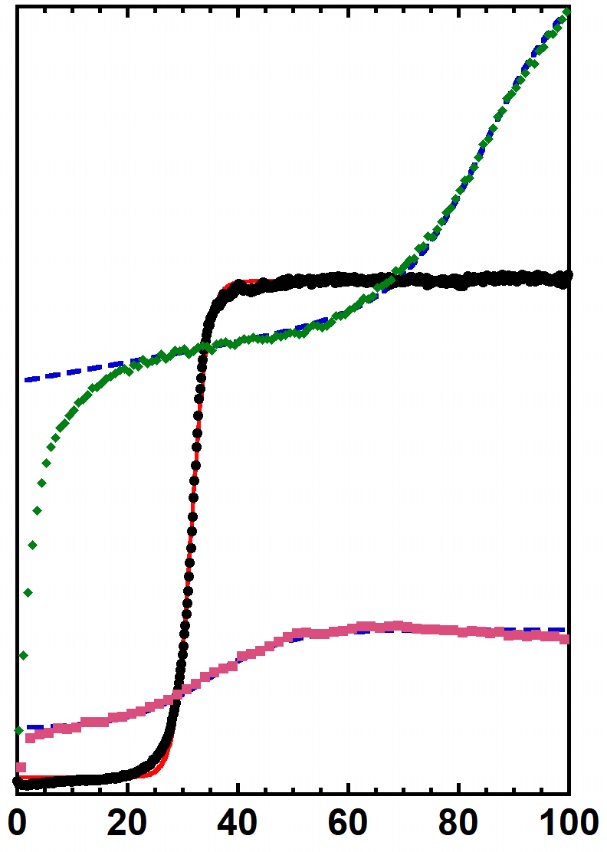
*

**Supplementary Figure S6.** Illustration of manual adjustment of parameters in equation **1.1** to reproduce the second sigmoidal process.
